# Anti-proliferative and anti-invasive effects of exogenous thermostable MnSOD in gastric cancer associated with p53 and ZEB1 expression

**DOI:** 10.7150/jca.102600

**Published:** 2025-03-03

**Authors:** Hailong Li, Hao Wang, Zong Li, Natalia Kelley, Matt Ouyang, Jia-Wei Wu, Fanguo Meng, Wen-Bin Ou

**Affiliations:** 1 Institute of Molecular Enzymology, School of Biology & Basic Medical Sciences, Suzhou Medical College of Soochow University, Suzhou, China.; 2 Zhejiang Provincial Key Laboratory of Silkworm Bioreactor and Biomedicine, College of Life Sciences and Medicine, Zhejiang Sci-Tech University, Hangzhou, China.; 3 Department of Pathology, Brigham and Women's Hospital and Harvard Medical School, Boston, MA, USA.; 4 Boston Latin School, Boston, MA, USA.

**Keywords:** gastric cancer, exogenous thermostable MnSOD, p53, ZEB1

## Abstract

The incidence of gastric cancer accounts for the first malignant tumor of the digestive tract. Although some progress in gastric cancer treatments has been made, uncontrollable drug resistance makes the development of new targeted drugs and treatment options increasingly urgent. The biological function of endogenous manganese superoxide dismutase (MnSOD) has been widely studied, whereas the anti-tumor growth effects of exogenous thermostable MnSOD in gastric cancer, an oral recombinant protein drug, are still unclear. Here, compared to normal gastric epithelial cell line and enzymatic dead mutant MnSOD H29A, we show that exogenous MnSOD treatment resulted in reduction of cell viability, colony formation, migration, and invasiveness; inhibition of SGC7901 xenograft growth; induction of apoptosis and arrest of G_2_-phase population in gastric cancer by an enzymatic activity-dependent manner; upregulation of p53, p21, and E-cadherin; and downregulation of cyclin D1 and N-cadherin. Unexpectedly, MnSOD treatment induced zinc finger E-box homeobox 1 (ZEB1) expression in SGC7901 gastric cancer cells, which was associated with a poor five-year survival rate and poor prognosis in gastric cancer patients. However, anti-proliferative effects of exogenous MnSOD were enhanced in SGC7901 after ZEB1 knockdown, whereas attenuated in BGC823 after ZEB1 restoration. These findings indicate that the exogenous thermostable MnSOD inhibited gastric cancer growth associated with p53 and ZEB1 expression levels and highlight that the exogenous thermostable MnSOD as an oral drug warrants evaluation as a novel therapeutic strategy in gastric cancer.

## Introduction

Gastric cancer is one of the leading causes of cancer-related death. According to the latest World Cancer Report 2020, the incidence (5.6%) and mortality (7.7%) of gastric cancer are ranked fourth and fifth among all cancer types, respectively 1. Most gastric cancers are stomach adenocarcinoma (STAD, 90-95%), and STAD is divided into two types: diffuse and intestinal according to its histopathological type 2. Due to the low detection rate, most patients are diagnosed at an advanced stage, so the overall prognosis of patients with gastric cancer is poor 3. Thus, it is imperative to identify new molecular targets and effective drug interventions in gastric cancer.

In recent years, various molecular mechanisms have been demonstrated to be closely related to the pathogenesis and drug resistance of gastric cancer. The major driver mutations of gastric cancers include *human epidermal growth factor receptor-2* (*HER2*) 4, *serine/threonine kinase 11* (*STK11*) 5, *E-cadherin* 6, *AT-rich interactive domain 1A* (*ARID1A*) 7, *Kirsten rat sarcoma virus* (*KRAS*) amplification 8, *A-T mutation* (*ATM*), *BRCA1 Interacting Protein 1* (*BRIP1*), *RAD51 paralog D* (*RAD51D*), or *PIK3CA* 9. Additionally, NF-κB signaling has been found to be one of the most frequently activated pathways in epithelial-mesenchymal transition (EMT) and metastasis in gastric cancer 10-12. Rho/Rho-associated protein kinase (ROCK) pathway also plays a key role in gastric cancer cell metastasis and invasion by recruiting IQ domain GTPase activating protein 1 (IQGAP1) 13-16, while IL-6 induces gastric cancer cell invasion by activating the c-Src/RhoA/ROCK signaling pathway 17.

Metalloenzyme superoxide dismutase (SOD), is divided into four types: manganese SOD (Mn-SOD), iron SOD (Fe-SOD), copper/zinc SOD (Cu/Zn-SOD), and nickel SOD (Ni-SOD) 18, 19. It has been reported that SOD has an important link with a variety of human health problems, including red blood cell-related diseases, fibrosis, post-cholecystectomy pain syndrome, malignant breast disease, steroid-sensitive nephrotic syndrome, amyotrophic lateral sclerosis, neuronal apoptosis, Alzheimer's disease, AIDS, and cancer 20-23. MnSOD plays a crucial role in the initiation, progression, and treatment of gastric cancer, with its actions exhibiting complexity 24-30. On the one hand, MnSOD can enhance the sensitivity of gastric cancer cells to the chemotherapeutic drug doxorubicin and inhibit tumor growth through oncolytic adenoviruses and other methods, demonstrating its tumor suppressive function 24,25. On the other hand, the gene polymorphisms of MnSOD are associated with the susceptibility to gastric cancer 26, and the increased expression levels of MnSOD are also related to the metastasis and invasive growth patterns of gastric cancer, suggesting that it may promote tumor development 27,28. In addition, MnSOD is also involved in regulating the level of oxidative stress and metabolic reprogramming within gastric cancer cells, further impacting the initiation and progression of tumors 29,30. Therefore, MnSOD may become a new target for the diagnosis and treatment of gastric cancer.

Supplementation with exogenous SOD has been reported to enhance the antioxidant defense of the host 31, and a large body of evidence gathered from clinical and animal models suggests that SOD is beneficial in a variety of applications, including reducing fibrosis after radiation therapy; preventing aging, diabetes, tumor formation and hepatitis C-related fibrosis 32 - 35; and reducing the cytotoxic and cardiotoxic effects of anticancer drugs 36. Due to its excellent antioxidant and therapeutic properties, various SOD products have been applied in the pharmaceutical, health care product, food additive, and cosmetic industries 37. However, SOD protein stability limited its practical application 38.

In the present study, we successfully developed a highly thermostable form of MnSOD derived from the thermophilic bacterium HB27, which is intended for use as an oral protein therapeutic or dietary supplement. This research represents a significant departure from previous studies by focusing on the efficacy of orally administered exogenous MnSOD. The current data found that the thermostable exogenous MnSOD exhibits potent anti-proliferative and anti-invasive properties against gastric cancer, both in *vitro* and in *vivo*.

## Materials and Methods

### Antibodies and reagents

Primary mouse monoclonal antibodies to p53 (sc-126) and cyclin D1 (sc-20044) were obtained from Santa Cruz Biotechnology (Santa Cruz, CA). Primary rabbit polyclonal antibodies to p21 (#2947) and ZEB1 (#3396) were from Cell Signaling Technology (Beverly, MA, USA). Anti-actin antibody (abs137975) was gained from Absin Bioscience Inc. (Shanghai, China). Trizol, Lipofectamine, and Plus reagent were purchased from Invitrogen Life Technologies (Carlsbad, CA, USA). The thermostable *MnSOD* wild type (WT) gene from thermus thermophilus HB27 was cloned. The MnSOD wild type (WT) and MnSOD H29A (Mn^2+^ binding site in SOD) mutation constructs were generated in pcDNA3, overexpressed in *E. coli*. BL21 stain and purified by using ion-exchange DEAE-Sepharose bead column. Purified MnSOD WT and mutant (H29A) were homogeneous on 10% SDS-PAGE. Lentiviral *ZEB1* shRNAs and *ZEB1* expression constructs were purchased from GeneCopoeia (Guangzhou, China).

### Cell lines and cell culture

Gastric cancer cell line SGC7901 is a moderately differentiated human lymph node metastatic gastric adenocarcinoma cell, BGC823 is a poorly differentiated gastric adenocarcinoma cell and RGM-1 cells are normal gastric mucosal epithelial cells. SGC7901 and BGC823 are kind gifts from Dr. Haibo Qiu at the Department of Gastric Surgery, Sun Yat-sen University Cancer Center, RGM-1 cells were purchased from Shanghai Yaji Biotechnology Co., Ltd. (NO. YS0059C). Cell lines were regularly screened for mycoplasma contamination using Mycoplasma Stain Assay Kit (Beyotime Biotechnology). SGC7901, RGM-1 and BGC823 cells were cultured in RPMI-1640 (Gibco) supplemented with 10% fetal bovine serum (FBS), 1% penicillin /streptomycin, and 1% L-glutamine in 5% CO_2_ at 37 °C.

### Preparations of* ZEB1* shRNA lentiviruses and virus infection

Lentivirus preparations were produced by co-transfecting LVRUG6P- or LV201.1-puro (empty vector, containing *ZEB1* shRNAs*,* or *ZEB1*-ORF construct), and helper virus packaging plasmids Δ8.9 and vsvg (at a 10:10:1 ratio) into HEK293T cells. Transfections were carried out using Lipofectamine and Plus reagent. Lentiviruses were harvested at 24, 36, 48, and 60 h post-transfection, and frozen at -80 °C in aliquots of appropriate amounts for single-use infection. Well-validated shRNAs were used for ZEB1 knockdown. The cells were seeded in six-well plates and lentiviral *ZEB1* shRNAs or *ZEB1* expressed construct infections were carried out in the presence of 10 ng/μL polybrene. All lentiviral experimental results were performed in duplicate. At least three independent assays were performed.

### Measurement of MnSOD enzymatic activity

In a water bath at 25℃, 2.35 mL 50 mM trimethylol aminomethane (#ASO1492-0500, Sangon Biotech.)-hydrochloric acid buffer solution (pH 8.2, containing 1 mM EDTA), 2 mL ddH_2_O, and 0.15 mL 45 mM Pyrogallol (#A606880-0100, Sangon Biotech) hydrochloric acid solution was added in sequence into a 10 mL colorimetric tube. After adding Pyrogallol hydrochloric acid solution, the solution was mixed immediately and poured into a colorimetric dish. The light absorption value of the initial point was measured and recorded. After 1 min at 325 nm, the absorption difference between the two values is the autooxidation rate of pyrocatechol ∆A_325_ (min^-1^). Finally, 20 μL of the dilution wild type MnSOD or mutated MnSOD (H29A) solution was added to inhibit the cautoxification rate of pyrocatechol at about ½ ∆A_325_ (min-1). Using the formula for Enzyme activity (U/mL) =
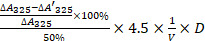
 (V: sample volume (mL), D: sample dilution multiples), quantitative analysis of MnSOD activity was carried out. In addition, the enzyme activity of MnSOD (100 μg/μL) was measured under the simulated gastric acid condition (pH 2.0) for 0.2, 0.5, 1.0, 2.0, and 4.0 h.

### Measurement of ROS levels

According to the manufacturer`s protocol, ROS levels were measured in SGC7901 and BGC823 cell lines using the Reactive Oxygen Species Assay Kit (Beyotime Biotechnology Co. Shanghai). Namely, the oxidative conversion of cell-permeable dichlorofluorescein diacetate (DCFH-DA) to fluorescent dichlorofluorescein (DCF) indicates changes in intracellular ROS levels. Briefly, SGC7901 and BGC823 cells in 6-well culture plates were incubated with 10 μM DCFH-DA at 37 °C for 20 min at 72 h post-treatment with MnSOD WT and H29A mutant protein. Then, the DCF fluorescence distribution of the cells was detected at a 488 nm excitation wavelength and a 535 nm emission wavelength by a fluorospectrophotometer (F4000, Japan) or at FITC channel by a flow cytometer (V6B5R3, Agilent) after the cells were washed twice with PBS. All assays were performed in triplicate. The data were analyzed and exported by NovoExpress (Agilent Technologies, lnc).

### Protein lysate preparations and western blotting

Protein lysates were prepared using lysis buffer (IP buffer) (1% NP-40, 50 mM Tris-HCl, pH 8.0, 100 mM sodium fluoride, 30 mM sodium pyrophosphate, 2 mM sodium molybdate, 5 mM EDTA and 2 mM sodium orthovanadate) containing protease inhibitors (10 μg/mL aprotinin, 10 μg/mL leupeptin and 1 mM PMSF) then rocked overnight at 4°C. Lysates were cleared by centrifugation at 15,000 rpm for 30 min at 4°C, and supernatant protein concentrations were determined using a Bio-Rad protein assay (Bio-Rad Laboratories, Hercules, CA). Electrophoresis and western blotting were performed as described previously 39. The hybridization signals were detected by chemiluminescence (Immobilon TM Western, Millipore Corporation, MA), and captured using a Tanon 5500 chemiluminescence imaging system.

### Cell viability analysis

SGC7901 and BGC823 cells were plated at 3000 cells per well in a 96-well flat-bottomed plate (Greiner, Frickenhausen, Germany). Proliferation analysis was carried out at 3 or 6 days after treatment with MnSOD WT, MnSOD H29A mutant, lentiviral *ZEB1* shRNAs, or lentiviral *ZEB1* in SGC7901 and BGC823 using the MTT assay, and quantified using a Microplate Reader (TECAN, Austria). The data were normalized to the empty vector group or H_2_O. All the assays were performed in triplicate wells, and at least three independent experiments were performed for each cell line.

### Colony-formation assay

Parental SGC7901 and BGC823 cells, the stably *ZEB1* silenced SGC7901, or the BGC823 cells with ZEB1 restoration were plated at 1000 cells/well in six-well plates and cultured until each clone consisted of 30-50 cells, and then the clones were treated with different concentrations of MnSOD WT. The cells were washed once with PBS, fixed in methanol for 20 min, and then stained with 0.1% crystal violet for 30 min. The excess stain was removed by washing with distilled water. Colonies were dissolved by using 900 µL 33% acetic acid solution and A_570_ was measured after colonies were photographed. The experiments were performed in duplicate wells and repeated three times.

### Cell cycle analysis

SGC7901 and BGC823 cells in six-well plates were trypsinised and washed with PBS buffer at 4 days post-treatment with MnSOD (50 U, 100 U, and 200 U). The cells were slowly added into pre-cooled 70% ethanol, mixed gently, and fixed overnight at 4°C. Nuclear staining was performed with a DAPI-containing PI solution and the cell suspension was immediately analyzed in a flow cytometer (BD Accuri C6, BD Biosciences). Data analysis was performed using Flow Jo and CFlow Plus. The experiments were performed in duplicate wells and repeated three times.

### Apoptosis assays

Apoptosis was evaluated using the PE Annexin V Apoptosis Detection Kit I (BD Pharmingen, San Jose, CA). SGC7901 and BGC823 cells in six-well plates were trypsinised and washed twice with cold PBS buffer at 4 days post-treatment with MnSOD (50 U, 100 U, and 200 U), then resuspended in 500 μL 1×Binding buffer. 100 μL of the cell suspension were removed and treated with 3 µL of PE Annexin V and 3 µL of 7-AAD for 15 min at 25 °C in the dark. When the time was up, 400 µL of 1× Binding buffer was added, shaken, and mixed well. The stained cells were analyzed in a flow cytometer (BD FACS Aria, Special Order System) within 2 h. CellQuest software (BD Biosciences) was used to analyze the data.

### Wound healing assays

Slashes were created in near-confluent SGC7901 and BGC823 cell cultures using the tip of a P-100 pipetman. Plates were photographed after treatment with MnSOD WT (10, 25, 50, 100, and 200 U) and MnSOD H29A mutant (10, 50, and 100 μg/μL) for 36 and 72 hours using a Leica DMI 3000B inverted microscope (Leica Microsystems, Germany). Experiments were performed in triplicate.

### Transwell Matrigel assays

Migration and invasiveness of gastric cancer cells were evaluated by the Matrigel assay. SGC7901 and BGC823 cells (2 × 10^4^) treated with MnSOD (50 U, 100 U, and 200 U) were suspended in 0.2 ml of SFM and seeded on the upper chamber of each well with 0.5 ml of 10% serum-containing medium added to the lower chamber. After 48 h, noninvasive cells that remained on the upper surface of the filter were removed using a cotton swab, and cells that remained adherent to the underside of the membrane were fixed in methanol and stained with 0.1% crystal violet. The membrane on the chamber was cut into a 96-well plate and decolorized with 33% acetic acid solution to read the OD value at 570 nm. Experiments were performed in triplicate.

### Xenotransplant murine models

Female adult athymic nude mice (6-8 weeks old) were housed in a specific pathogen-free facility. Mice were injected subcutaneously at bilateral armpits with 1x10^6^ SGC7901 cells suspended in BD Matrigel. Five mice were treated once daily for 3 weeks with MnSOD WT (375 U/g) by intraperitoneal injection. The others (n =5) were maintained for 3 weeks with water. Mice were sacrificed by cervical dislocation and necropsied to evaluate tumor volume and weight. The studies were conducted in accordance with recognized ethical guidelines (U.S. Common Rule), were approved by Zhejiang Sci-Tech University Institutional Review Boards.

### Statistical analysis

Student's t-tests were performed on data from cells treated with MnSOD/shRNAs/*Lenti*-*ZEB1* or H_2_O/LVR/LV201 empty vector (control). Statistically significant differences between control and treatment were defined as *p < 0.05, **p < 0.01, ***p < 0.001, and ****p < 0.0001.

## Results

### Characterization of pH-stability of the purified MnSOD WT, and ROS level of gastric cancer cells after treatment with exogenous purified MnSOD WT or H29A mutant

Under the same protein concentration (100 µg/µL) at pH 7.0, enzymatic activity of WT and H29A mutant was measured. Activity of MnSOD WT and mutant was 58.8 × 10^3^ U/mL and 1.6 × 10^3^ U/mL, respectively.

**Figure 1:**
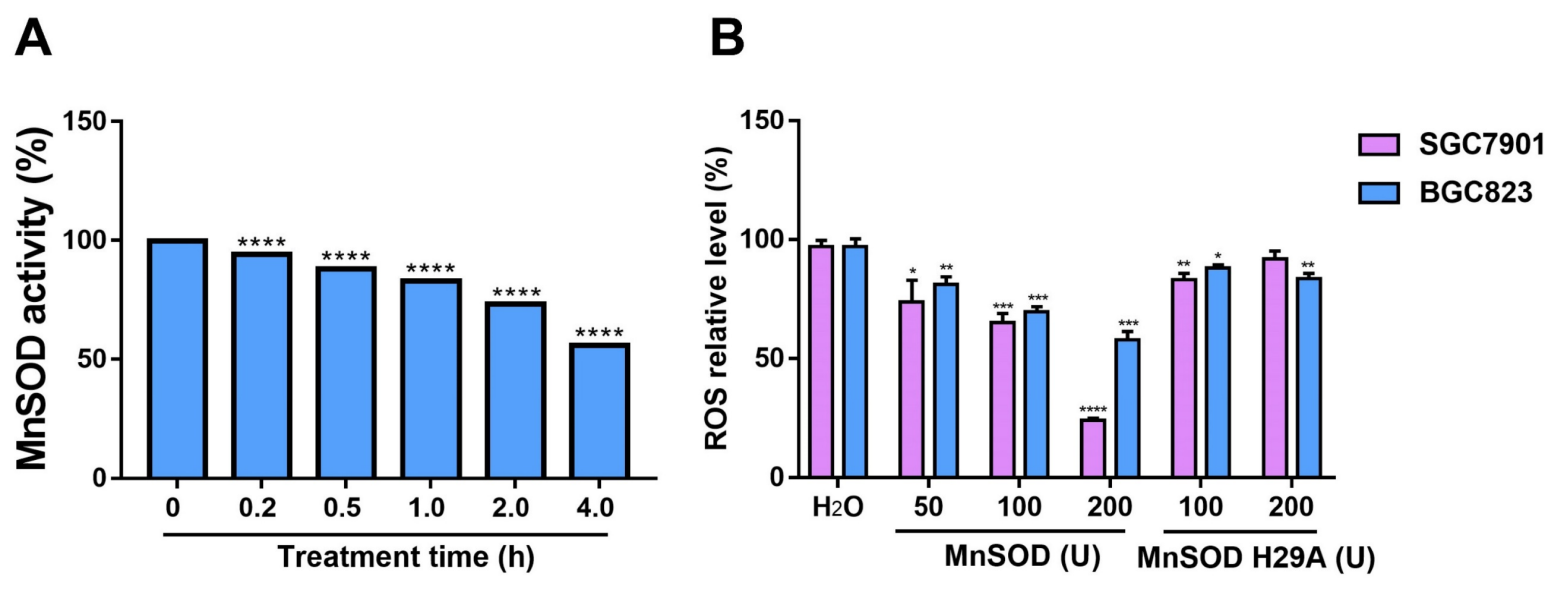
Characterization of pH-stability of purified MnSOD WT and ROS levels of gastric cancer cell lines after treatment with exogenous purified MnSOD WT and/or H29A mutant. **A**) The enzymatic activity of the purified MnSOD WT (100 U) from BL21 cells was determined under simulated gastric acid conditions (pH 2.0) for 0.2, 0.5, 1.0, 2.0, and 4.0 h. Statistically significant differences are presented as ****p < 0.0001. **B**) ROS levels were measured in SGC7901 and BGC823 gastric cancer cell lines using the Reactive Oxygen Species Assay Kit at 72 hours post-treatment with exogenous purified MnSOD WT and H29A mutant protein. All assays were performed in triplicate. Statistically significant differences between the control and MnSOD WT/H29A mutant are presented as *p < 0.05, **p < 0.01, ***p < 0.001, and ****p < 0.0001.

The enzyme activity of the purified MnSOD WT was measured under simulated gastric acid conditions (pH 2.0) for 0.2, 0.5, 1.0, 2.0, and 4.0 h (Figure 1A). Compared with the control group at 0 h, low pH treatment resulted in a decrease of MnSOD enzymatic activity in a time-dependent manner, and the enzyme activity remained at 60% at pH 2.0 condition for 4 hours (Figure 1A). Additionally, the ROS levels were also measured in gastric cell lines (SGC7901 and BGC823) after treatment with different concentrations of MnSOD WT and H29A mutant (Figure 1B). MnSOD WT treatment resulted in a 70% and 40% decrease in ROS levels in SGC7901 and BGC823 cells, respectively, whereas MnSOD H29A mutant had little effect on ROS generation in these cells (Figure 1B).

### Exogenous MnSOD WT treatment significantly inhibits proliferation, colony formation, and induces apoptosis in gastric cancer cells

**Figure 2:**
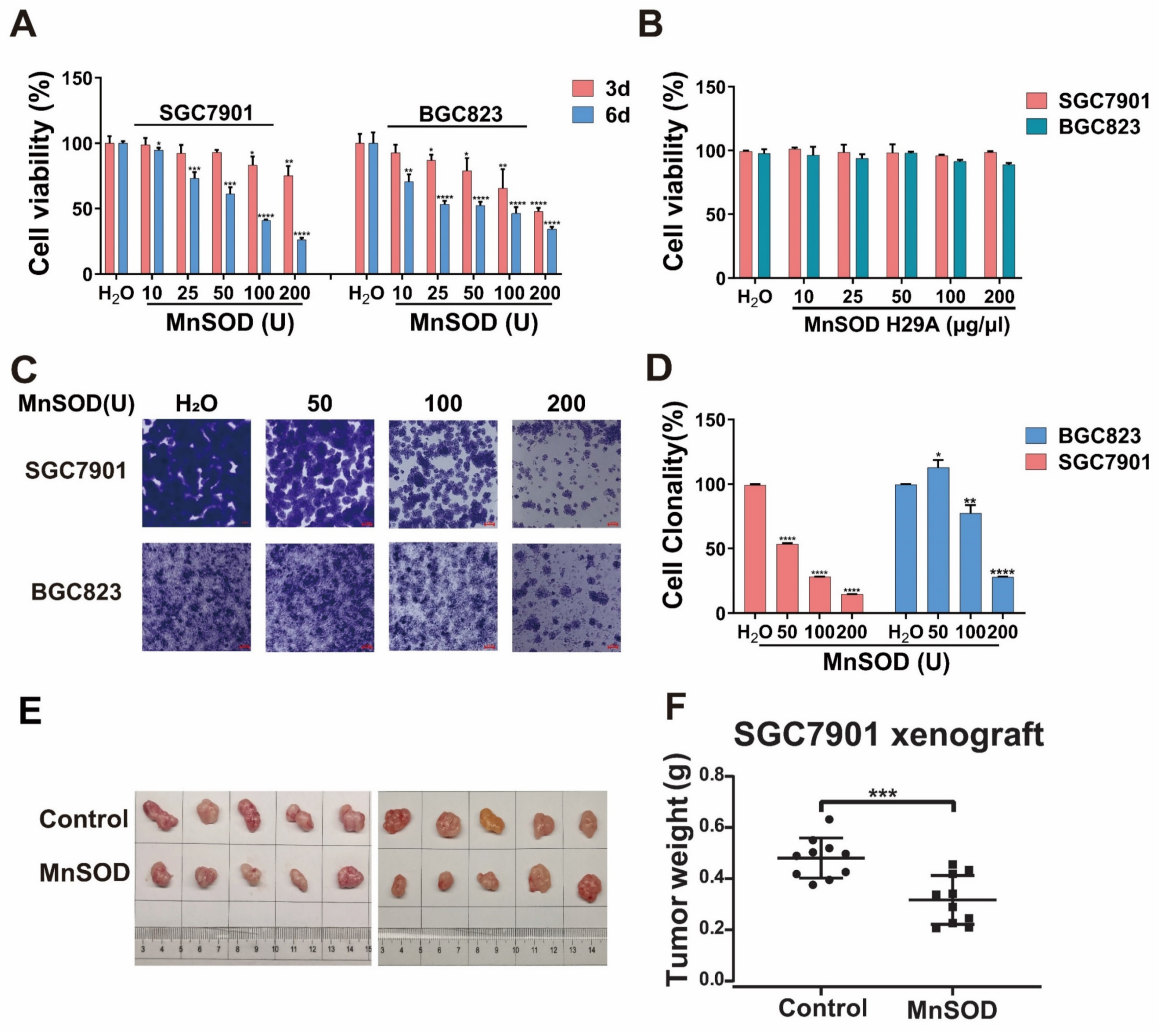
Exogenous MnSOD treatment inhibits proliferation, colony formation, migration, and invasiveness; and induces apoptosis in gastric cancer cells. **A**) Cell viability was evaluated by an MTT assay at days 3 and 6 in SGC7901 and BGC823 cell lines after treatment with MnSOD WT. Data represent the mean values (±s.d.) of quadruplicate cultures averaged from two independent experiments and normalized to the H_2_O control. Statistically significant differences between H_2_O control and MnSOD treatment are presented as *p < 0.05, **p < 0.01, ***p < 0.001, ****p < 0.0001. **B**) Cell viability was evaluated by an MTT assay at day 6 in SGC7901 and BGC823 cell lines after treatment with MnSOD H29A mutant. Data represent the mean values (±s.d.) of quadruplicate cultures averaged from two independent experiments and normalized to the H_2_O control. **C**) MnSOD WT treatment inhibited colony growth in SGC7901 and BGC823. Colony growth experiments were performed in triplicate. Scale bars: 400 μm. **D**) Quantitation (A_570_) of SGC7901 and BGC823 cell colony growth after treatment with MnSOD WT. Statistically significant differences between H_2_O control and MnSOD treatments are presented as *p < 0.05, **p < 0.01, ****p < 0.0001. **E**) MnSOD WT treatment (375 U/g, daily) inhibited SGC7901 xenograft growth. **F)** MnSOD WT treatment inhibited SGC7901 xenograft weight. Statistically significant differences between H_2_O control and MnSOD WT treatment are presented as ***p < 0.001.

Anti-proliferative and pro-apoptotic effects of exogenous MnSOD WT were evaluated in SGC7901 and BGC8203 cell lines by cell viability, colony formation, apoptosis, and cell cycle assays. MnSOD WT treatment inhibited cell proliferation in a dose-dependent manner (Figure 2A), whereas treatment with H29A mutant had little effect on cell viability in these cells (Figure 2B), and using RGM-1 cells as a control, it had only a minimal impact on cellular viability (Figure S1A). Compared with H_2_O control, 200 U MnSOD WT treatment reduced viability at day 6 by 70% in SGC7901 and by 60% in BGC823, respectively (Figure 2A). Colonies of gastric cancer cells treated with MnSOD WT were inhibited when compared with H_2_O-treated cells (Figure 2C). Relative to the control, colony formation decreased by approximately 80% in SGC7901 cells and 70% in BGC823 cells after treatment with 200 U MnSOD WT (Figure 2D). Further, MnSOD WT treatment inhibited the growth of SGC7901 xenografts in mice, with reductions in xenograft size and weight compared to the control xenografts (Figure 2E and 2F).

**Figure 3:**
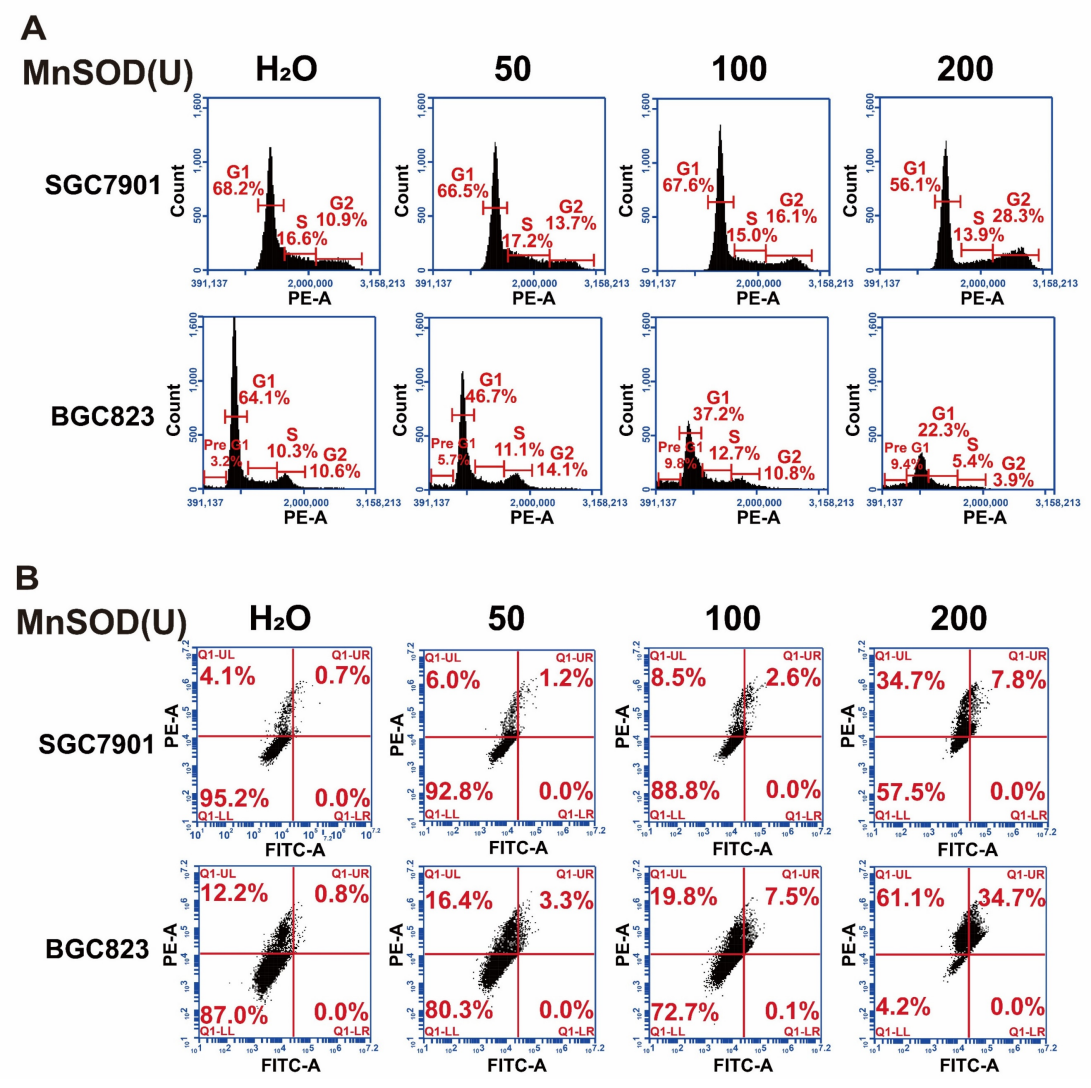
Exogenous MnSOD treatment induces apoptosis in gastric cancer cells.** A**) Cell cycle analysis was performed in SGC7901 and BGC823 at day 4 post-treatment with MnSOD WT. Cell cycle experiments were performed in triplicate. **B**) Apoptosis assays were performed in SGC7901 and BGC823 at day 4 post-treatment of MnSOD WT by using the PE Annexin V Apoptosis Detection Kit I. Apoptosis experiments were performed in triplicate.

MnSOD WT treatment in SGC7901 resulted in a decrease of G_1_-phase population, from 68.2% in the control-treated cells to 56.1% in 200 U MnSOD-treated cells, which was accompanied by an increase in the G_2_-phase population from 10.9% with the control to 28.3% with MnSOD WT (Figure 3A and Supplementary table 1). Additionally, MnSOD WT induced an increase in apoptotic cells at 4 days of treatment in BGC823 by pre-G_1/0_ phase cell population analysis, from 3.2% in the control cells to 9.4% in 200 U MnSOD-treated cells (Figure 3A). In apoptosis assays, treatment with MnSOD WT induced greater apoptosis in SGC7901 and BGC823 cells in a concentration-dependent manner (200 U MnSOD: 42.5% and 91.7%, respectively) than treatment with the contrast (Figure 3B, Supplementary table 2), MnSOD had a minimal effect on the cell cycle and apoptosis of RGM-1 cells (Figure S1B and S1C).

### Exogenous MnSOD WT treatment blocks migration and invasiveness in gastric cancer cells

**Figure 4:**
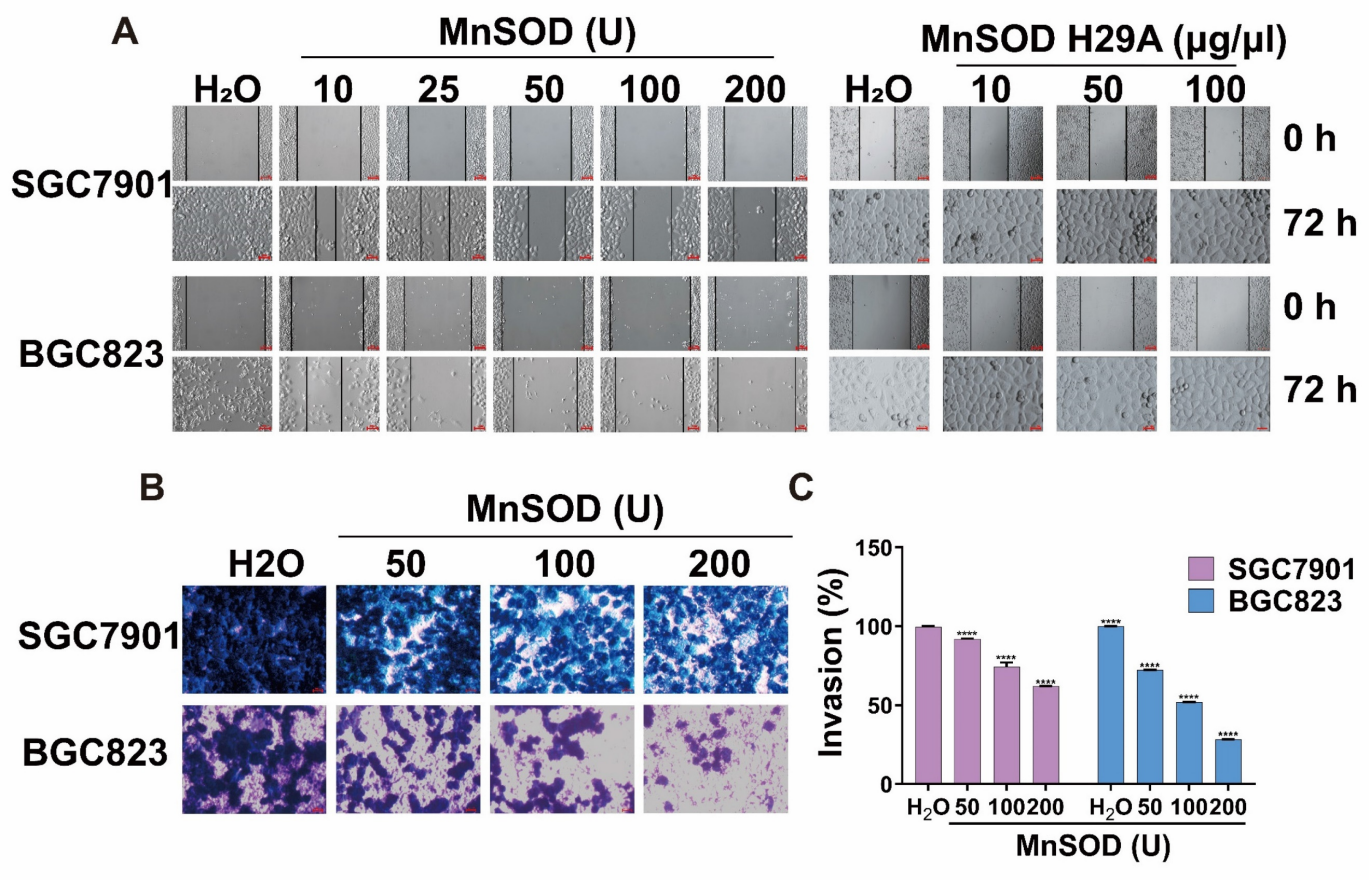
Exogenous MnSOD WT treatment inhibits migration and invasiveness in gastric cancer cells. **A**) *In vitro* wound healing assays reveal that treatment with MnSOD WT significantly inhibits the migration of gastric cancer cells, whereas the MnSOD H29A mutant treatment exhibits minimal inhibition of wound closure in SGC7901 and BGC823 cell lines. Scale bars: 100 μm. **B**) Transwell migration assays show that MnSOD WT treatment effectively inhibits migration and invasion of gastric cancer cells (SGC7901 and BGC823). Scale bars: 400 μm. Transwell experiments were performed in triplicate. **C**) Quantitation (A_570_) of cell invasiveness in SGC7901 and BGC823 at day 2 post-treatment with MnSOD WT. Statistically significant differences between H_2_O control and MnSOD WT treatments are presented as ****p < 0.0001.

Migration and invasion assays in SGC7901 and BGC823 cells were performed by using wound healing and transwell Matrigel after treatment with MnSOD WT and/or H29A mutant. Wound-healing assays demonstrated that MnSOD WT impaired wound closure in a dose-dependent manner at 72 hours in SGC7901 and BGC823, treatment with H29A mutant had little impact on wound closure in these cells, whereas complete wound closure was observed in H_2_O control cells (Figure 4A). Transwell Matrigel assays demonstrated inhibition of gastric cell invasiveness in a dose-dependent manner after treatment with MnSOD WT (Figure 4B), with about 10-40% suppression in SGC7901 and about 25-75% suppression in BGC823, respectively, as compared with the control (Figure 4C).

### Exogenous MnSOD WT treatment up-regulates p53 and p21, and down-regulates cyclin D1 and EMT marker in gastric cancer cells

**Figure 5:**
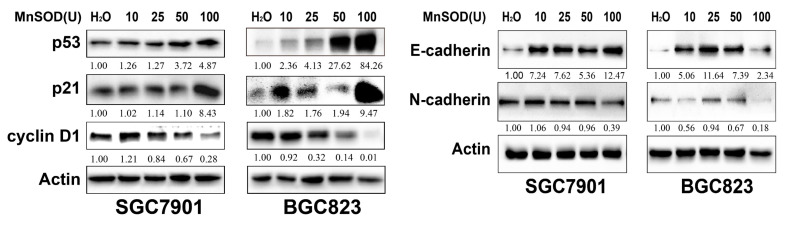
Immunoblot evaluation of the expression of cell cycle checkpoints (p53, p21, and cyclin D1) and EMT markers (E-cadherin and N-cadherin) in SGC7901 and BGC823 cells at 96 hours post-treatment with MnSOD WT. Actin stains are lane loading control. The grouping of blots cropped from different parts of the same gel. The number at bottom of each stain box indicates quantitation of protein expression.

The expression of cell cycle checkpoints (p53, p21, and cyclin D1) and EMT markers (E-cadherin and N-cadherin) in SGC7901 and BGC823 cells at 96 hours post-treatment with MnSOD WT was evaluated by immunoblot (Figure 5). MnSOD WT treatment induced expression of p53, p21, and E-cadherin, and inhibited expression of cyclin D1 and N-cadherin in both cell lines in a concentration-dependent manner (Figure 5).

### High ZEB1 expression indicates poor prognosis in gastric cancer patients

**Figure 6:**
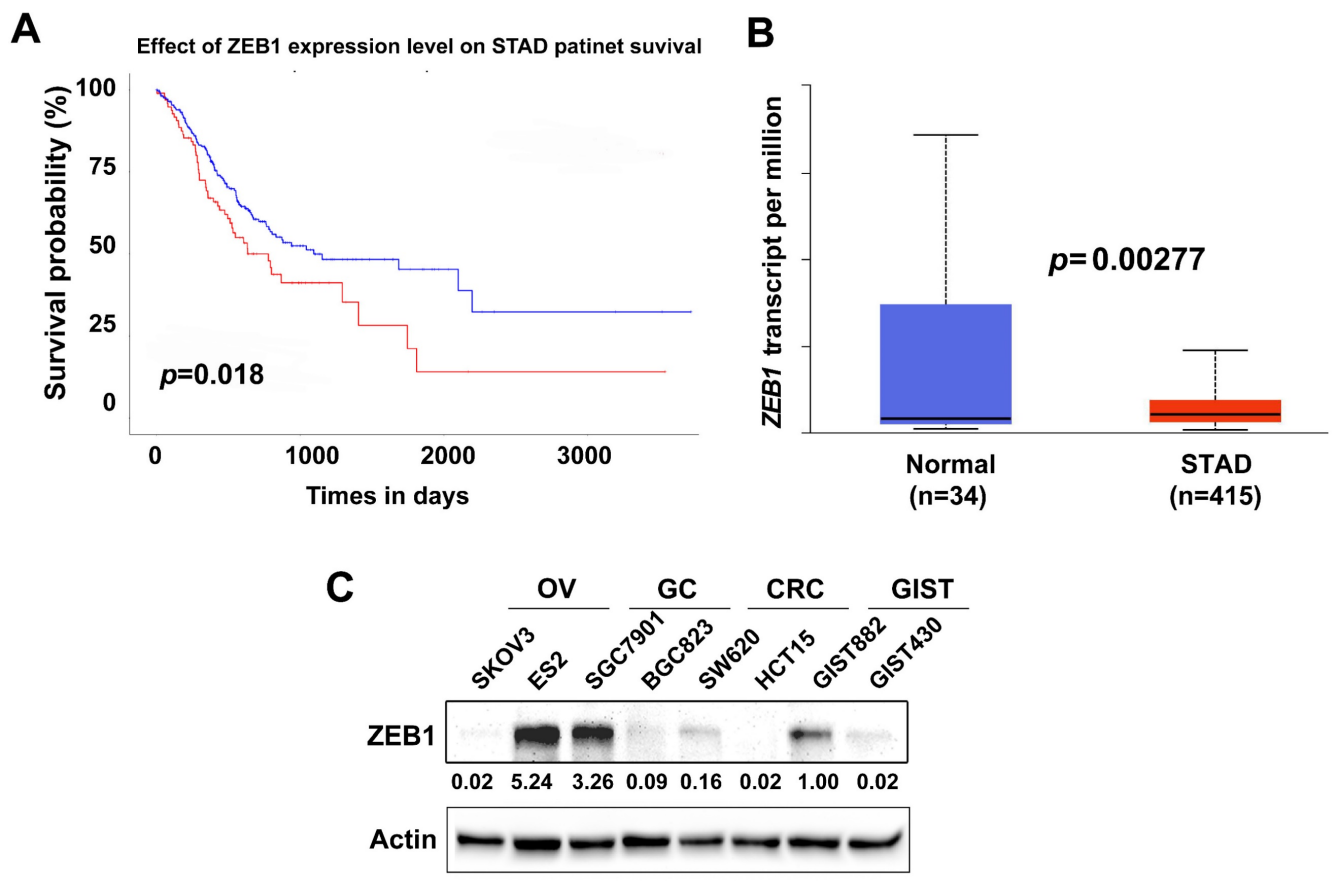
High ZEB1 expression indicates poor prognosis in gastric cancer patients.** A**) Survival analysis of TCGA STAD dataset demonstrates that high ZEB1 expression level correlates with poor overall survival (*p* = 0.018) in STAD patients. Red line indicates high ZEB1 expression (n=99); Blue line indicates low-medium ZEB1 expression (n=293). **B**) TCGA gene expression profiling data analysis for 415 STAD shows that ZEB1 expression is significantly higher in STAD patient samples compared to adjacent non-neoplastic tissue samples (*p* = 0.00277).** C)** Immunoblotting evaluation of ZEB1 expression in ovarian cancer cell lines (SKOV3 and ES2), gastric cancer cell lines (SGC7901 and BGC823), colorectal cancer lines (SW620 and HCT15), and gastrointestinal stromal tumor cell lines (GIST882 and GIST430). Actin stain is a loading control. The grouping of blots cropped from different parts of the same gel.

High ZEB1 expression has been indicated to promote tumor growth in gastric cancer 40. Herein, using the Cancer Genome Atlas (TCGA) stomach adenocarcinoma (STAD) dataset (Ualcan.path.uab.edu/analysis), we first analyzed the prognostic value of ZEB1 expression in STAD patients. High ZEB1 expression in STAD was correlated with reduced overall survival (*p* = 0.018) (Figure 6A). Compared with adjacent normal tissues (n=34), ZEB1 was significantly overexpressed in STAD (n=415) (*p* = 0.00277) (Figure 6B). ZEB1 expression was further evaluated by immunoblotting in two ovarian cancer (OV) cell lines (SKOV3 and ES2), two gastric cancer (GC) lines (SGC7901 and BGC823), two colorectal cancer (CRC) lines (SW620 and HCT15), and two gastrointestinal stromal tumor (GIST) cell lines (GIST882 and GIST430). Immunoblotting showed that ZEB1 was overexpressed in SGC7901 and ES2; weakly expressed in BGC823, SW620, GIST882, and GIST430; and undetectable in SKOV3 and HCT15 (Figure 6C).

### Anti-proliferative and -migration effects of exogenous MnSOD WT treatment are modulated by ZEB1 expression in gastric cancer cells

**Figure 7:**
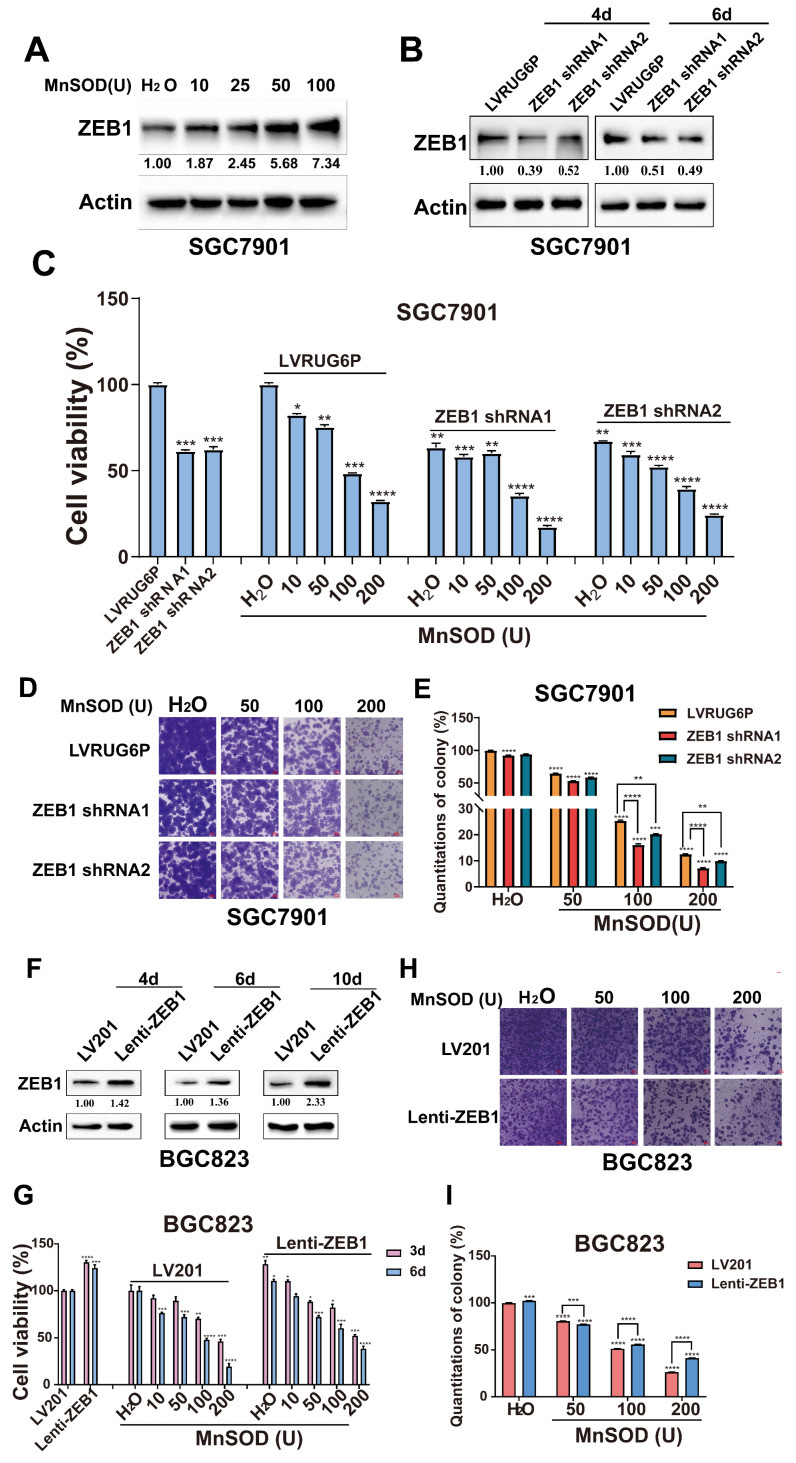
Anti-proliferative and -migration effects of exogenous MnSOD WT treatment are associated with ZEB1 expression in gastric cancer cells.** A**) Immunoblot evaluation of ZEB1 expression in SGC7901 at day 4 post-treatment with MnSOD WT. Actin stain is a loading control. The grouping of blots cropped from different parts of the same gel. **B**) Immunoblot evaluation of ZEB1 expression in SGC7901 at 4- and 6-days post-infection with *ZEB1 shRNAs*. Actin stain is a loading control. The grouping of blots cropped from different parts of the same gel. **C**) Cell viability was evaluated by MTT assay in SGC7901 after treatment with *ZEB1 shRNA1/2* and MnSOD WT individually or in combination for 6 days. Data represent the mean values (±s.d.) of quadruplicate cultures averaged from two independent experiments and were normalized to the empty lentivirus infections and/or H_2_O. Statistically significant differences between LVR/H_2_O control and shRNAs or MnSOD WT treatment are presented as *p < 0.5, **p < 0.01, ***p < 0.001, ****p < 0.0001. All the assays were performed from triplicate experiments. **D**) Coordinated treatment with *ZEB1 shRNA1/2* and MnSOD WT more markedly reduced colony growth in SGC7901 than either treatment alone. Scale bars: 400 μm. **E**) Quantitation of colony formation in SGC7901 at 7 days after *ZEB1 shRNA1/2* and MnSOD WT treatments. Statistically significant difference is indicated as **p < 0.01, ****p < 0.0001. **F**) Immunoblot evaluation of ZEB1 expression in BGC823 at days 4, 6, and 10 post-infections with *Lenti-ZEB1*. Actin stain is a loading control. The grouping of blots cropped from different parts of the same gel. **G**) Cell viability was evaluated by MTT 3 and 6 days after treatment with MnSOD WT and *Lenti-ZEB1*. Data were normalized to LV201 control and represent mean values (± s.d.) from quadruplicate cultures averaged from two independent experiments for each cell line. Statistically significant differences between LV201 control and *Lenti-ZEB1* construct infections are presented as *p < 0.05, **p < 0.01, ***p < 0.001, and ****p < 0.0001. **H**) Colony growth assays were performed after treatment with MnSOD WT for 7 days. Colony growth experiments were performed in triplicate. MnSOD WT treatment after ZEB1 restoration led to a less reduction in colony formation in BGC823 than either intervention alone. Scale bars: 400 μm. **I**) Quantitation of cell colony growth in BGC823 with ZEB1 restoration after treatment with MnSOD. Statistically significant differences between LV201 + H_2_O control and *Lenti-ZEB1* + MnSOD are presented as ***p < 0.001 and ****p< 0.0001.

We investigated the effects of exogenous MnSOD WT treatment on ZEB1 expression in SGC7901 cells. Immunoblotting showed that MnSOD WT treatment resulted in an increase of ZEB1 expression in SGC7901 in a dose-dependent manner (Figure 7A). Furthermore, ZEB1 expression was silenced by lentivirus-mediated shRNAs in SGC7901. The immunoblotting evaluation showed that shRNA knockdown resulted in about a 50%-60% reduction of ZEB1 expression (Figure 7B). Compared with empty vector LVR, ZEB1 silencing reduced cell viability at day 6 by ~ 40% in SGC7901 (Figure 7C) and colony formation (Figure 7D and 7E).

Additive anti-proliferative effects were demonstrated by viability and colony formation after combined treatment with *ZEB1 shRNAs* and exogenous MnSOD WT in SGC7901 (Figure 7C-7E). As compared to the H_2_O control in the LVR group, MnSOD WT treatment (10, 50, 100, and 200 U) at day 6 reduced cell viability by 20%-65%. Coordinated treatment with *ZEB1 shRNAs* and MnSOD WT reduced viability by 45%-80% for SGC7901 (Figure 7C). Furthermore, combinatorial treatment with *ZEB1 shRNAs* and MnSOD WT resulted in greater inhibition of colony growth in SGC7901, as compared to either treatment alone. Compared to the control, colony growth reduced by ~ 20% after ZEB1 knockdown, by ~ 40%-85% after MnSOD WT treatment (50, 100, and 200 U), and by ~ 45%-92% under coordinated treatment with *ZEB1 shRNAs* and MnSOD WT (Figure 7D and 7E).

We next investigated the impact of ZEB1 restoration in BGC823 cells on the antiproliferative effects of MnSOD WT treatment. Immunoblotting showed that ZEB1 expression was restored in BGC823 by lentiviral infection with *Lenti*-*ZEB1* (Figure 7F). ZEB1 restoration resulted in a ~ 25% increase in cell viability in BGC823, as compared with the same cells after infection with the Lenti-LV201 control (Figure 7G). Likewise, ZEB1 restoration promoted colony formation (Figure 7H), resulting in ~ 5% increased colony formation in BGC823 (Figure 7I). Furthermore, ZEB1 restoration attenuated inhibition of proliferation and colony formation by MnSOD WT in BGC823 cells (Figure 7G-7I). Compared to the control, cell viability and colony growth increased by ~ 25% and 5% after ZEB1 restoration. Proliferation and colony formation were inhibited by ~ 80% and 75% after MnSOD WT treatment (200 U), whereas both showed inhibition by ~ 60% and 60% under treatment with MnSOD WT after ZEB1 restoration, respectively (Figure 7G-7I).

## Discussion

Gastric cancer remains important worldwide with incidence and mortality ranking fifth and fourth in the world, respectively 1. The combination of platinum compounds with 5-Fu chemotherapy, the first line therapy, has improved symptoms and extended survival 41. Addition of a third drug treatment, such as docetaxel or doxorubicin, has resulted in increased toxicity but improvement of patient survival 42. Various novel immunotherapies and molecular targeted therapies, such as anti-HER2, also significantly increase the survival of cancer patients 43. Although neo-adjuvant and adjuvant treatment strategies have markedly improved survival in patients with locally advanced gastric cancer (AGC), patient 5-year survival rate is still very low 44, and even with novel chemotherapy protocols and biological therapies, median overall survival (OS) is still less than 1 year 1. Therefore, the development of novel and more effective pharmacological interventions and therapeutic targets is required.

Our study stands out in the field by focusing on the application of an orally administered highly stable exogenous protein drug, MnSOD, for the treatment of gastric cancer. The most critical characteristic of such a protein drug is its stability against the extremely acidic conditions of the stomach. To this end, we conducted a novel evaluation of MnSOD enzymatic activity under simulated gastric acid conditions, a key aspect that differentiates our research from previous studies. Thus, we first evaluated MnSOD enzymatic activity under simulated gastric acid conditions. Enzyme activity remained ~ 60% after incubation for 4 hours at pH 2.0 (Figure 1A), indicating that the purified MnSOD from thermus thermophilus HB27 still harbors partial enzymatic activity and function in the human stomach. Additionally, H29 amino acid residue localizes to the Mn^2+^ binding site in SOD H29A mutant resulted in inactivation of MnSOD and attenuated the antigenerative effects of MnSOD on ROS in SGC7901 and BGC823 cells (Figure 1B). This finding provides critical insight into the structural determinants of MnSOD function, which has not been previously explored in the context of gastric cancer treatment.

Previous studies have extensively investigated the clinic impact and potential mechanisms of endogenous MnSOD in a wide range of human malignancies, including gastric cancer 24-30. Weak MnSOD expression levels in gastric cancer are significantly correlated with poor patient survival 45. MnSOD overexpression results in increased resistance of gastric carcinoma cells to DOX 46. Additive anti-proliferative effects are shown in gastric cancer after combination inhibition of MnSOD and NFKB signaling. Co-targeting of MnSOD and NFκB signaling restores the efficacy of DOX treatment in DOX-resistant gastric cancer cells 47. Furthermore, the combination of recombinant oncolytic adenoviruses containing plasminogen Kringle 5 mutant and MnSOD significantly inhibited gastric cancer growth 48.

Although endogenous MnSOD function has been evaluated in gastric cancer, exogenous thermostable MnSOD function remains unclear. In the current report, MnSOD WT treatment significantly inhibited cell viability, colony formation, wound healing, and Matrigel transwell invasiveness; blocked cell cycle at G_2_-phase; induced cell apoptosis in gastric cancer cells; and inhibited SGC7901 xenograft growth (Figure 2, 3, and 4) which was associated with induction of p53 and p21 expression, down-regulation of cyclin D1, and inhibition of EMT (upregulation of E-cadherin and downregulation of N-cadherin) (Figure 5). In contrast, inactivated MnSOD H29A mutant had minimal anti-proliferative or anti-migration effects on these cells (Figure 2, 3, and 4), indicating that the anti-proliferative and pre-apoptotic effects of exogenous thermostable MnSOD WT depend on its enzyme activity. These findings highlight the use of exogenous thermostable MnSOD in the form of an oral protein drug as a novel therapeutic strategy for gastric cancer treatment.

Zinc finger E-box-binding homeobox 1 (ZEB1, previously known as TCF8) is a zinc finger and homeodomain transcription factor. Various studies have shown that ZEB1 has an oncogenic role and functions as an EMT regulator in gastric cancers. ZEB1 expression is significantly higher in gastric carcinoma tissue than in adjacent normal mucosa. Patients with strong ZEB1 expression had significantly poorer survival than those with weak expression. ZEB1 overexpression is related to the occurrence and development as well as invasion and metastasis of gastric carcinoma 33, 49, 50. ZEB1 knockdown inhibited ubiquitin ligase CUL4A- and Wnt5a- driven proliferation, EMT, and metastasis in gastric cancer 51, 52. Expression of the DNA endonuclease Mus81 positively correlated with ZEB1 expression in gastric cancer, and Mus81 promotes gastric metastasis by regulating the *ZEB1* transcription 53. LINC01559 stabilizes *ZEB1* mRNA and upregulates ZEB1 expression in gastric cancer cells through recruiting insulin like growth factor 2 mRNA binding protein 2 54. Bcl2-associated athanogene 4 regulates EMT, invasion, and metastasis of gastric cancer cells by activation of the PI3K/AKT/NF-κB/ZEB1 axis 55. The current data shows that MnSOD WT treatment induces ZEB1 expression in a dose-dependent manner (Figure 7A). Based on previous studies, we speculate that the anti-proliferative effects of MnSOD WT partially depend on ZEB1 expression levels. Therefore, we investigated the cell viability and colony formation under treatment with MnSOD after ZEB1 shRNA knockdown in SGC7901 and ZEB1 restoration in BGC823, respectively (Figure 7). Additive anti-proliferative effects of MnSOD were observed in SGC7901 after ZEB1 shRNA knockdown (Figure 7A-7D), whereas anti-proliferative effects of MnSOD were attenuated in BGC823 after ZEB1 restoration (Figure 7E-7H).

The novelty of the current study is multifaceted: Firstly, we demonstrated for the first time the feasibility of orally administering a highly stable exogenous MnSOD protein in the field of gastric cancer therapy; secondly, the exogenous thermostable MnSOD protein maintains its stability under physiological conditions, representing a significant breakthrough compared to previous studies that primarily investigated endogenous MnSOD; finally, our study reveals a novel mechanism of exogenous MnSOD, wherein the therapeutic effects are mediated through the regulation of ZEB1 to induce the tumor suppressors p53 and p21. We are confident that these results fill a gap in the existing literature and offer a promising new approach for the treatment of gastric cancer. Our research focuses on the potential therapeutic application of exogenous thermostable MnSOD as a treatment for gastric cancer, an area that has not been extensively explored.

The current research did not include human subjects or surgically obtained human gastric cancer tissue samples after treatment with exogenous thermostable MnSOD, which limits the direct translation of our laboratory findings to the human in vivo status. The inclusion of such samples would indeed provide valuable insights into the clinical relevance of our work. While we appreciate the importance of direct clinical correlation, we must acknowledge that our study did not involve human subjects or clinical tissue samples due to the complex ethical and practical considerations associated with clinical trials involving exogenous MnSOD treatment. Thus, our data highlight this as an area for future research, emphasizing the need for studies that can bridge the gap between laboratory findings and clinical applications.

## Conclusion

Our studies demonstrate that exogenous thermostable MnSOD WT inhibits gastric cancer growth and invasiveness. These effects are enhanced by co-treatment with ZEB1 shRNA *in vitro* and *in vivo*. Our studies show that the anti-proliferative effects of MnSOD WT are partially modulated by ZEB1 expression levels. These findings further define strategies for gastric cancer therapeutics exploration in future studies.

## Supplementary Material

Supplementary figure and tables.
