# Supplementary material for: Anti-proliferative and anti-invasive effects of exogenous thermostable MnSOD in gastric cancer associated with p53 and ZEB1 expression
Source: J Cancer. 2025 Mar 3;16(6):2062–74. doi: 10.7150/jca.102600 (PMC11905419; doi:10.7150/jca.102600)
Supplement: Supplementary file 1 — Supplementary figure and tables. [file jcav16p2062s1.pdf]

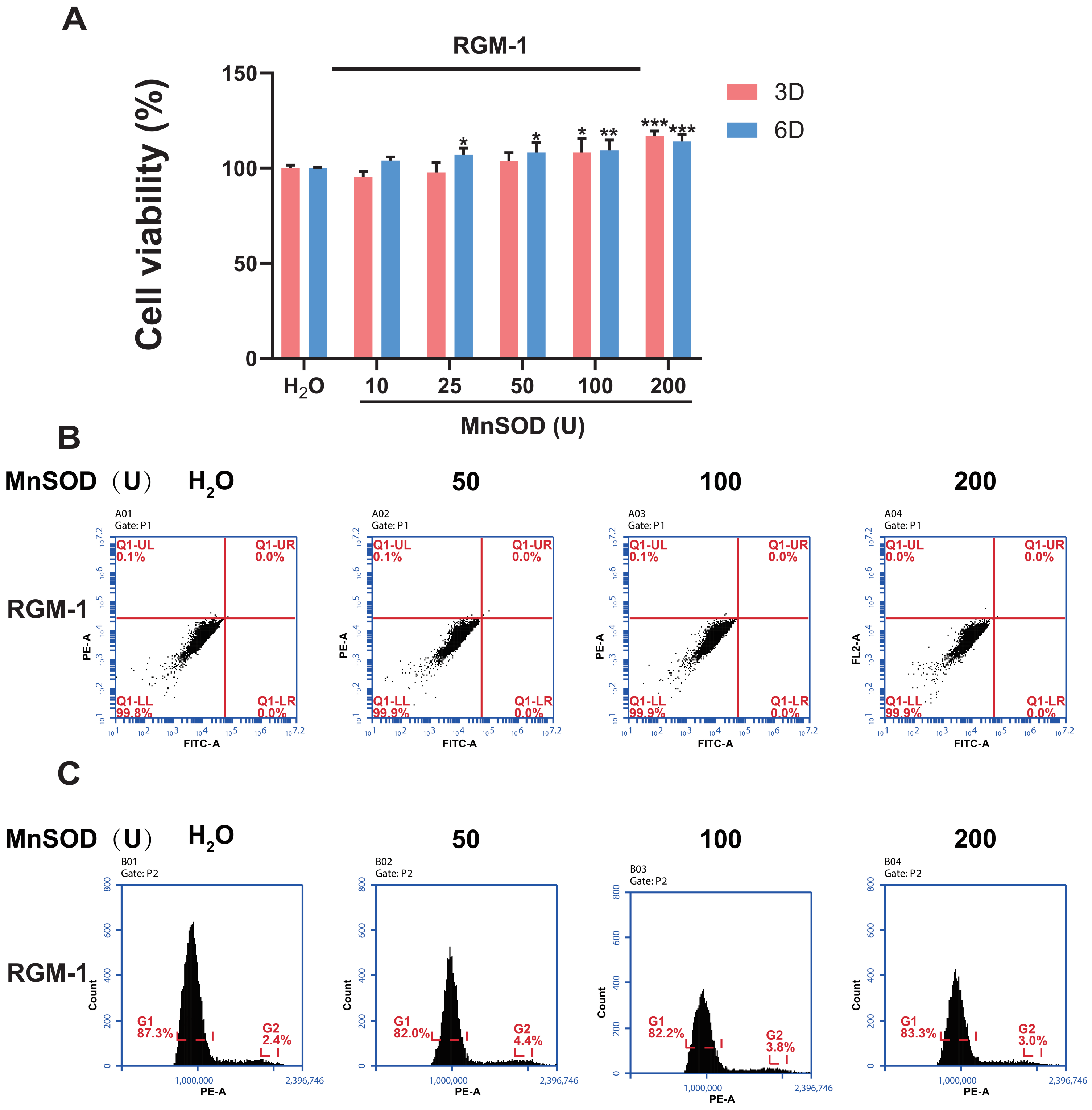


**Figure S1**. Effects of MnSOD treatment on the proliferation, cell cycle, and apoptosis of RGM-1 cells. **A)** Cell viability was evaluated by an MTT assay at days 3 and 6 in RGM-l cell lines after treatment with MnSOD WT. Data represent the mean values (±s.d.) of quadruplicate cultures averaged from two independent experiments and normalized to the $H_2O$ control. Statistically significant differences between $H_2O$ control and MnSOD treatment are presented as *$p < 0.05$, **$p < 0.01$, ***$p < 0.001$, ****$p < 0.0001$. **B)** Apoptosis assays were performed in RGM-1 at day 4 post-treatment of MnSOD WT by using the PE Amexin V Apoptosis Detection Kit I. Apoptosis experiments were performed in triplicate. **C)** Cell cycle analysis was performed in RGM-1 at day 4 post-treatment with MnSOD WT. Cell cycle experiments were performed in triplicate.

**Supplementary table1.** Cell cycle analyses (%), as shown in Figure 3A, after MnSOD treatment.

| **MnSOD** | **SGC7901** | | | | **BGC823** | | | |
|---|---|---|---|---|---|---|---|---|
| (**U**) | **Pre-$G_1$/$G_0$** | **$G_1$** | **S** | **$G_2$** | **Pre-$G_1$/$G_0$** | **$G_1$** | **S** | **$G_2$** |
| **$H_2O$** | 4.7 | 68.2 | 16.2 | **10.9** | **15.0** | 64.1 | 10.3 | 10.6 |
| **50** | 2.6 | 66.5 | 17.2 | **13.7** | **27.5** | 46.7 | 11.1 | 14.7 |
| **100** | 1.3 | 67.6 | 15.0 | **16.1** | **39.3** | 37.2 | 12.7 | 10.8 |
| **200** | 1.7 | 56.1 | 13.9 | **28.3** | **68.4** | 22.3 | 5.4 | 3.9 |

**Supplementary table 2**. Cell apoptosis analyses (%), as shown in Figure 3B, after MnSOD treatment.

| **MnSOD** | **SGC7901** | | | | **BGC823** | | | |
|---|---|---|---|---|---|---|---|---|
| (**U**) | **Q1** | **Q2** | **Q3** | **Q4** | **Q1** | **Q2** | **Q3** | **Q4** |
| **$H_2O$** | 4.1 | 0.7 | 95.2 | 0 | 8.1 | 0.9 | 91.0 | 0 |
| **50** | 6.0 | 1.2 | 92.8 | 0 | 11.2 | 2.4 | 86.4 | 0 |
| **100** | 8.5 | 2.6 | 88.8 | 0 | 13.3 | 5.5 | 81.1 | 0.1 |
| **200** | 34.7 | 7.8 | 57.5 | 0 | 54.3 | 37.4 | 8.3 | 0 |
